# A molecular map of long non-coding RNA expression, isoform switching and alternative splicing in osteoarthritis

**DOI:** 10.1093/hmg/ddac017

**Published:** 2022-01-28

**Authors:** Georgia Katsoula, Julia Steinberg, Margo Tuerlings, Rodrigo Coutinho de Almeida, Lorraine Southam, Diane Swift, Ingrid Meulenbelt, J Mark Wilkinson, Eleftheria Zeggini

**Affiliations:** Technical University of Munich (TUM), School of Medicine, Munich 81675, Germany; Institute of Translational Genomics, Helmholtz Zentrum München-German Research Center for Environmental Health, Neuherberg 85764, Germany; Institute of Translational Genomics, Helmholtz Zentrum München-German Research Center for Environmental Health, Neuherberg 85764, Germany; Daffodil Centre, University of Sydney, a joint venture with Cancer Council NSW, Sydney, NSW 1340, Australia; Department of Biomedical Data Sciences, Section Molecular Epidemiology, Leiden University Medical Center, Leiden 2333 ZC, The Netherlands; Department of Biomedical Data Sciences, Section Molecular Epidemiology, Leiden University Medical Center, Leiden 2333 ZC, The Netherlands; Institute of Translational Genomics, Helmholtz Zentrum München-German Research Center for Environmental Health, Neuherberg 85764, Germany; Department of Oncology and Metabolism, University of Sheffield, Metabolic Bone Unit, Sorby Wing Northern General Hospital Sheffield, Sheffield, S5 7AU, UK; Department of Biomedical Data Sciences, Section Molecular Epidemiology, Leiden University Medical Center, Leiden 2333 ZC, The Netherlands; Department of Oncology and Metabolism, University of Sheffield, Metabolic Bone Unit, Sorby Wing Northern General Hospital Sheffield, Sheffield, S5 7AU, UK; Institute of Translational Genomics, Helmholtz Zentrum München-German Research Center for Environmental Health, Neuherberg 85764, Germany; Technical University of Munich (TUM) and Klinikum Rechts der Isar, TUM School of Medicine, Munich 81675, Germany

## Abstract

Osteoarthritis is a prevalent joint disease and a major cause of disability worldwide with no curative therapy. Development of disease-modifying therapies requires a better understanding of the molecular mechanisms underpinning disease. A hallmark of osteoarthritis is cartilage degradation. To define molecular events characterizing osteoarthritis at the whole transcriptome level, we performed deep RNA sequencing in paired samples of low- and high-osteoarthritis grade knee cartilage derived from 124 patients undergoing total joint replacement. We detected differential expression between low- and high-osteoarthritis grade articular cartilage for 365 genes and identified a 38-gene signature in osteoarthritis cartilage by replicating our findings in an independent dataset. We also found differential expression for 25 novel long non-coding RNA genes (lncRNAs) and identified potential lncRNA interactions with RNA-binding proteins in osteoarthritis. We assessed alterations in the relative usage of individual gene transcripts and identified differential transcript usage for 82 genes, including *ABI3BP*, coding for an extracellular matrix protein, *AKT1S1*, a negative regulator of the mTOR pathway and *TPRM4*, coding for a transient receptor potential channel. We further assessed genome-wide differential splicing, for the first time in osteoarthritis, and detected differential splicing for 209 genes, which were enriched for extracellular matrix, proteoglycans and integrin surface interactions terms. In the largest study of its kind in osteoarthritis, we find that isoform and splicing changes, in addition to extensive differences in both coding and non-coding sequence expression, are associated with disease and demonstrate a novel layer of genomic complexity to osteoarthritis pathogenesis.

## Introduction

Osteoarthritis is a joint disease characterized by progressive degeneration of articular cartilage, remodeling of the underlying bone, and synovitis (1,2). It is the most common joint disorder and a major cause of pain and disability worldwide (3). Currently, no curative treatments are available, and management strategies focus on symptom alleviation through pain relief and joint replacement surgery, stressing the need to identify new targets. The defining hallmark of osteoarthritis progression is cartilage degeneration. Identification of transcriptomic changes in osteoarthritis can help elucidate genes and pathways that play a role in disease pathogenesis.

Previous studies have focused on gene-level changes in expression with larger sample sizes being required for an exhaustive characterization of differences (4–19). Recently, alterations in gene expression during osteoarthritis have also been attributed to epigenetic phenomena and their interaction (4,6,9–11,16–19). To assess the role of long non-coding RNA genes (lncRNAs), which have low expression levels, deep RNA sequencing (RNA-seq) is needed. To date, the majority of RNA-seq studies in osteoarthritis have explored expression differences at the gene level only, without considering the dynamics in the expression of multiple related transcripts. Alterations in relative transcript abundances or isoform-switches have been shown to play a role in other diseases (20,21). Disentangling the different isoforms is crucial as they can result in functionally different protein products, affect topology and mRNA stability (20). Differential use of untranslated transcripts and non-principal isoforms is primarily responsible for tissue-specific isoform expression patterns, with even minor alterations in isoform usage potentially having a significant impact on protein expression (20–22). Additionally, alternative splicing can affect function without inducing significant changes in expression.

**Figure 1:**
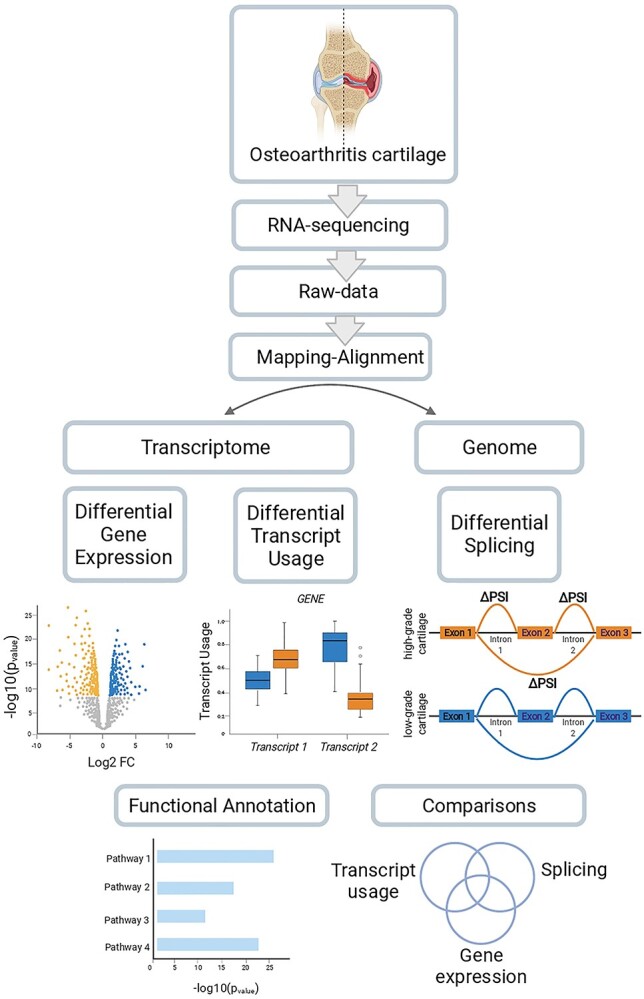
Study overview.

In this work, we investigated transcriptomic differences between low-osteoarthritis grade and high-osteoarthritis grade cartilage in 124 patients undergoing total knee replacement (Supplementary Material, Table S1) increasing the sample size by 50% compared to the largest study of its kind (5). We identified differentially expressed genes and performed extensive comparison with transcriptomic studies published to date to define an osteoarthritis-specific transcriptomic signature. We examined previously understudied biotypes (lncRNAs) (23), found enrichment for disease-involved biological pathways, and detected transcriptome-wide differences in isoform usage and alternative splicing to our knowledge for the first time (Fig. 1).

## Results

### Gene-level changes between low-osteoarthritis grade and high-osteoarthritis grade cartilage

**Figure 2:**
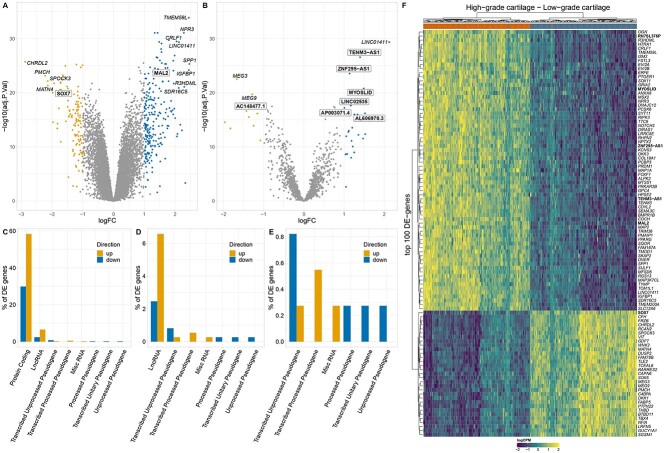
Differentially expressed (DE) genes between paired low- and high-osteoarthritis grade cartilage. (**A**) Differential expression of all genes. (**B**) Differential expression of lncRNA genes. Gene names shown in white boxes in A and B highlight newly implicated differentially expressed genes. (**C**) Biotype annotations of the DE genes. (**D**, **E**) Enlarged C. gene biotype bar plots showing less abundant biotypes. (**F**) Hierarchical clustering on top 100 DE genes (logCPM: log-counts-per-million). Gene names highlighted in bold show newly implicated DE genes.

In order to detect the most informative gene expression changes in osteoarthritis cartilage, we defined differentially expressed (DE) genes as those that had a larger than 2-fold (|log_2_FC| > 1) increased or decreased expression in high-osteoarthritis grade cartilage at 5% FDR. This resulted in 365 DE genes. Of these genes, 241, were significantly upregulated (log_2_FC:[1, 2.47]) and 124 were downregulated (log_2_FC:[−2.88, −1]) in high-osteoarthritis grade cartilage (Fig. 2A, Supplementary Material, Table S2). Comparison with 16 studies reporting osteoarthritis-specific alterations in cartilage tissue indicated large agreement among top signals and added 54 novel associations (4–19) (Fig. 2A and B, Supplementary Material, Table S2). *TMEM59L* (log_2_FC = 2.45, *P* = 1.4 × 10^−34^) was the most significantly upregulated gene. *TMEM59L* encodes a neuron-specific transmembrane protein mediating oxidative stress-induced cell death through caspase-3 in mice, a mechanism also associated with chondrocyte cell death in osteoarthritis experimental models (24,25). Conversely, *CHRDL2* (log_2_FC = −2.87, *P* = 1.8 × 10^−26^) was the most significantly downregulated gene, and the gene with the largest observed fold-decrease in expression levels in high-osteoarthritis grade compared to low-osteoarthritis grade cartilage. *CHRDL2* codes for chordin-like protein two, which is implicated in the negative regulation of cartilage formation (26).

**Figure 3:**
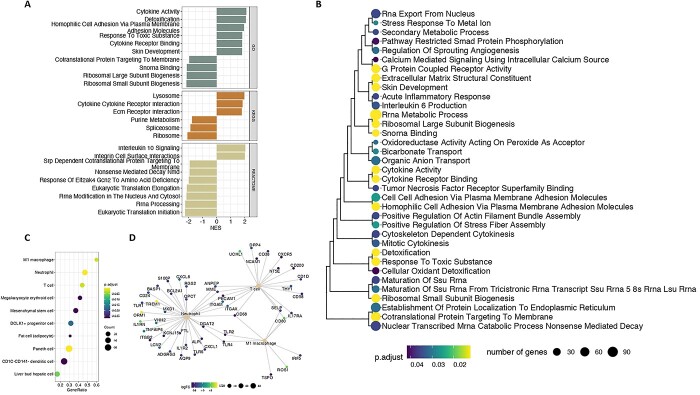
Gene set enrichments among DE genes. (**A**) Gene sets significantly associated with DE genes at 5% FDR. Only the ten gene sets with the most significant enrichment in each category are shown. Log_2_FC: log-fold-change. Adj. *P* val: FDR. (**B**) Hierarchical clustering of Gene Ontology (GO) terms based on gene semantic similarity using the Jaccard coefficient. (**C**) Gene set enrichments among cell type makers. (**D**) Genes associated with different inflammation-related cell types discussed in the text. Genes are colored according to their log_2_FC.

Following the identification of DE genes, we performed gene set enrichment analysis (GSEA) to identify coordinated changes in expression of biological pathways. The main pathways enriched were relevant to inflammation, extracellular matrix organization and the translation machinery (Supplementary Material, Table S3). Upregulated genes were mainly enriched for processes related to inflammation, including increased cytokine activity, neutrophil degranulation, signaling by GPCR; as well as structural terms including collagen formation, extracellular matrix organization and integrin interactions (Fig. 3A and B). The pathway with the highest positive enrichment (normalized enrichment score—NES) was ‘cytokine activity’ (NES = 2.12, *P* = 1.2 × 10^−2^) and the one that was most significantly upregulated was ‘integrin cell surface interactions’ (NES = 2.01, *P* = 5.4 × 10^−3^). The leading-edge subset (core genes) of the cytokine activity pathway included the upregulation of *SPP1* (osteopontin)*,* several members of interleukin families 1 and 6 (*CRLF1*, *IL1RN*, *IL36G*, *IL11*, *IL1B*) and members of the tumor necrosis factor family (*TNFSF18*, *TNFSF8*) indicating inflammation driven by mainly IL-1 and IL-6 cytokine families in osteoarthritis cartilage. Apart from increased expression of pro-inflammatory cytokines, we also observed upregulation of the interleukin-10 anti-inflammatory signaling pathway (NES = 2.04, *P* = 9.6 × 10^−3^). The leading edge of this pathway implicated upregulation of *IL1RN1* that encodes the interleukin 1 receptor antagonist. However, in contrast, the gene encoding IL10 itself was downregulated in our dataset (logFC = −0.74, *P* = 1.9 × 10^−7^), a finding which is consistent with the complex pattern of crosstalk between the different cytokines in osteoarthritis cartilage. In addition to that, we identified upregulation of the cell-type markers of M1 macrophages (NES = 2.07, *P* = 2.1 × 10^−3^), neutrophils (NES = 2.15, *P* = 3.2 × 10^−5^) and T cells (NES = 2.02, *P* = 2.1 × 10^−3^) indicating potential immune cell infiltration in osteoarthritis cartilage (Fig. 3C and D, Supplementary Material, Table S3). The leading-edge of ‘integrin cell surface interactions’ pathway included the upregulation of *SPP1* along with multiple collagen coding genes (*COL18A1*, *COL1A1*, *COL5A3*, *COL3A1*, *COL1A2*) indicating the extensive remodelling of the extracellular matrix. We identified downregulation (NES < 0) for spliceosome and RNA processing terms and terms relevant to ribosome formation and the translational machinery (Fig. 3A and B, Supplementary Material, Table S3). The leading edge of translation terms implicated suppression of several genes coding for ribosomal proteins (*RPS5*, *RPS15, RPS8, RPS6*, *RPL3*, *RPL38*). Such proteins are essential for maintaining the overall ribosomal subunit structures and this finding is indicative of aberrations in ribosome assembly in high-osteoarthritis grade cartilage (27). We identified positive enrichment for terms relevant to cholesterol metabolism, including the synthesis of bile acids and 27 hydroxycholesterols supporting the metabolic spectrum component of osteoarthritis (28). Upregulated DE genes were further enriched for targets of ZNF-507 transcription factor, which is predicted to interact with ADAMTS-7, a metallopeptidase previously associated with osteoarthritis (29,30) (Supplementary Material, Table S3).

### LncRNAs involved in osteoarthritis progression

**Figure 4:**
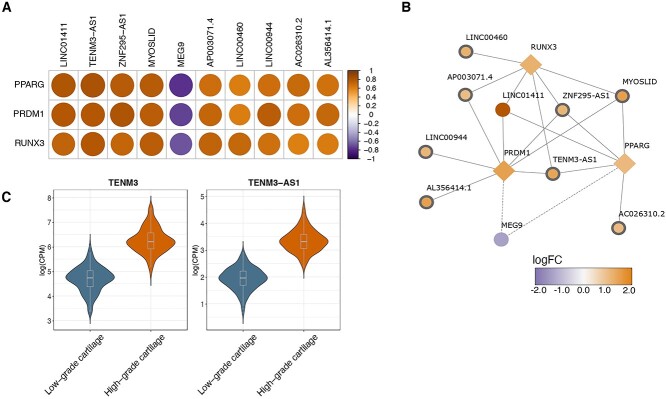
Differentially expressed lncRNAs between low- and high-osteoarthritis grade cartilage. (**A**) Heatmap of Spearman correlations between differentially expressed RBP genes and differentially expressed lncRNAs. (**B**) Network of differentially expressed lncRNAs targeted by three differentially expressed RBP genes that have been previously associated in osteoarthritis cartilage. Novel lncRNAs identified in our analysis are highlighted with a thicker border width. All network nodes are colored according to the log_2_FC: log-fold-change. (**C**) Expression (covariate-adjusted logCPM: log counts per million) of long noncoding RNA (lncRNA) *TENM3-AS1* and gene *TENM3* in low- and high-osteoarthritis grade cartilage. Both *TENM3-AS1* and *TENM3* were significantly upregulated in high-osteoarthritis grade cartilage. Violin plots show the expression distribution across samples. Boxplots center at the median and whiskers extend to 1.5 times the interquartile range.

Sequencing depth and mapping to non-coding transcriptome allowed us to further investigate lncRNAs. In total, we detected 33 lncRNAs among DE genes out of which 25 were reported for the first time in osteoarthritis cartilage (Supplementary Material, Fig. S1). The lncRNA with the largest increase was *MYOSLID* (log_2_FC = 1.49, *P* = 1.6 × 10^−21^), which has been reported to be in a positive feedback loop with the transforming growth factor (TGF)-β/SMAD pathway in vascular smooth muscle cells (31). The most significant newly implicated lncRNA was *TENM3-AS1* (log_2_FC = 1.38, *P* = 2.7 × 10^−27^), which was upregulated in high-osteoarthritis grade cartilage. The direction of effect was consistent with its sense gene *TENM3* (log_2_FC = 1.57, *P* = 3.3 × 10^−26^) indicating potential *cis* activity or co-expression due to its location proximity (Fig. 4C).

To further characterize DE lncRNAs, we examined their enrichment among RNA-binding proteins (RBPs), which have been recently recognized as important lncRNA regulators in disease (32,33). Differentially expressed lncRNA genes were overrepresented among 166 RBPs (downstream interaction data derived from LncSEA database (34)) (Supplementary Material, Table S4). Three out of the 166 enriched RBPs (*PRDM1*, *RUNX3* and *PPARG)* were also DE in our dataset and have been extensively linked to osteoarthritis (4–6,8,35–37). In order to detect RBPs that are potential regulators of lncRNAs and identify potential interactions, we examined the correlation of expression between the DE RBP genes and DE lncRNAs. We identified 20 significant correlations (*r* > 0.7) including 10 individual lncRNAs (Fig. 4A), eight out of which were associated for the first time with osteoarthritis (Supplementary Material, Tables S2 and S5). The highest positive correlation was found for the pairs *TENM3-AS1*-*PPARG* and *LINC01411-PPARG* and *ZNF295-AS1-PRDM1* (*r* > 0.8, *P* < 2e^−16^) (Fig. 4B). *PPARG* has been proposed as a therapeutic target for osteoarthritis (37), therefore its regulation axis could provide possible intervention points. Notably, we also detected a strong inverse correlation between *MEG9* and all of the DE RBP genes (*r* < 0.7, *P* < 2e^−16^) in our dataset.

### Transcript-level changes in osteoarthritis cartilage

**Figure 5:**
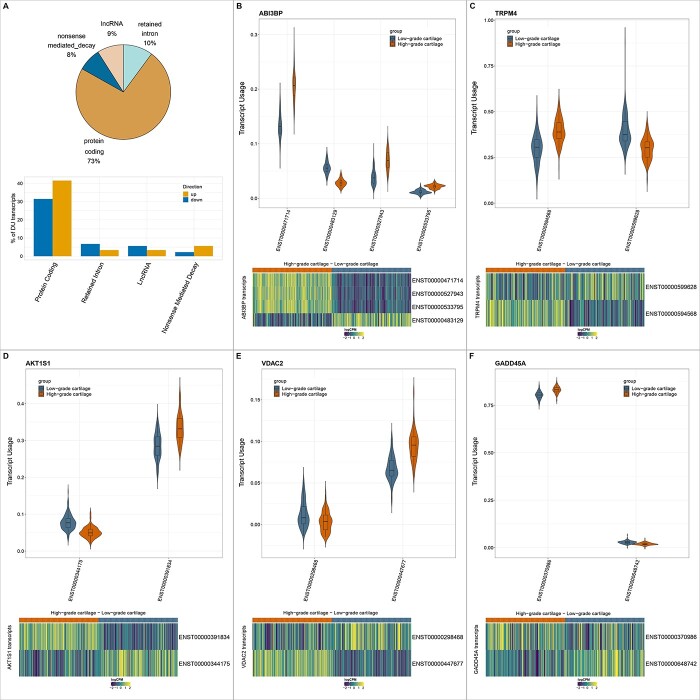
Differential transcript usage between low- and high-osteoarthritis grade cartilage. (**A**) Distribution of differentially used transcripts between low- and high-osteoarthritis grade cartilage among ENSEMBL transcript biotypes. (**B–F**) Violin plots show the distribution of usage of differentially used transcripts for *ABI3BP*, *TRPM4, AKT1S1, VDAC2* and *GADD45A*. Boxplots within the violin plots have their center at the median and whiskers extend to 1.5 times the interquartile range. Heatmaps show the normalized expression of the respective transcripts between low and high-osteoarthritis grade cartilage accounting for technical variation. logCPM: log-counts-per-million.

In order to gain a better understanding of the spliceosome and RNA processing terms enriched among downregulated DE genes, we examined fine changes in the balance of different isoforms by performing differential transcript usage (DTU) analysis. We identified 89 isoforms belonging to 82 genes that were differentially used between low- and high-osteoarthritis grade cartilage at 5% FDR (Supplementary Material, Table S6). The differentially used transcripts were 73% protein-coding, 10% included retained introns, 9% were annotated as lncRNAs and 8% were prone to non-sense-mediated decay (Fig. 5A). For *ABI3BP, AKT1S1, TRPM4, VDAC2* and *GADD45A*, we detected two or more significantly differentially used transcripts (isoform-switches) (Fig. 5B–F).

**Figure 6:**
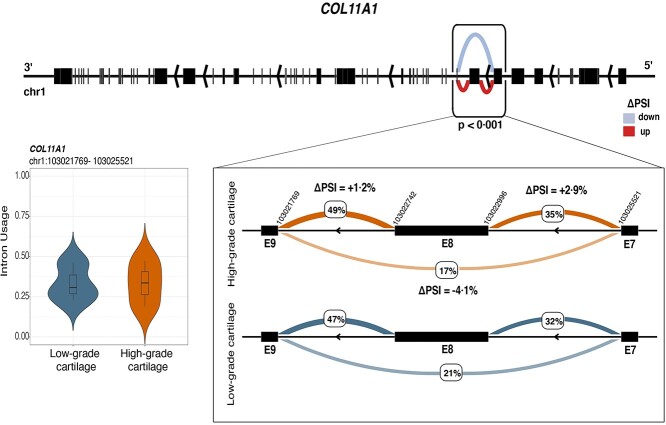
Differential splicing between low- and high-osteoarthritis grade cartilage for *COL11A1*. The figure illustrates decreased skipping of exon 8 of *COL11A1* in high-osteoarthritis grade cartilage. The exons affected by the skipping event fall within the N-terminal variable region of collagen. The violin plot shows the distribution of intron usage (covariate-adjusted PSI) of the differentially excised intron cluster containing exon 8 (E8) for low- and high-osteoarthritis grade cartilage.

**Figure 7:**
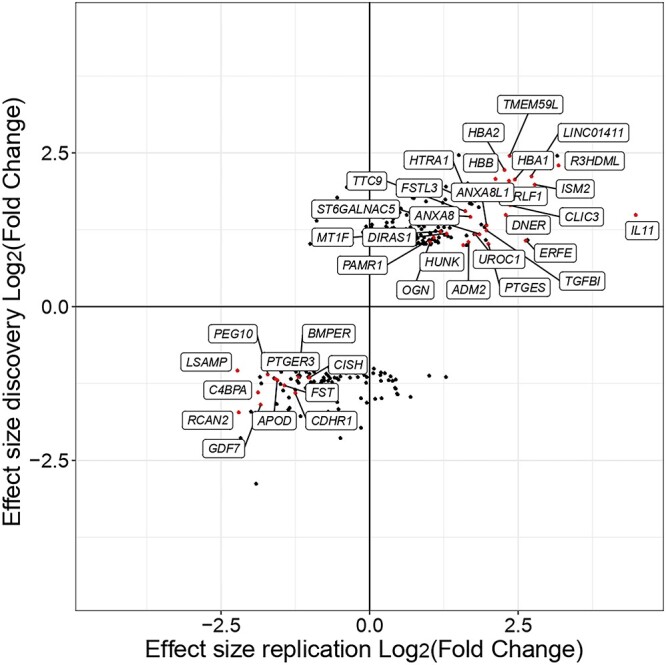
Comparison of gene expression differences of the DE genes identified in the discovery and replication datasets. The plot shows gene-level log-fold-changes of DE genes identified in the discovery compared to the replication dataset. Individual genes are shown as single points, and the color corresponds to whether the gene is identified as DE in discovery and replication dataset (red), in the discovery dataset only (black).

Among the leading signals, we detected four protein-coding transcripts of *ABI3BP*, a gene coding for an extracellular matrix protein and implicated in cell–extracellular matrix interactions (38). Transcripts *ENST00000471714* (log_2_FC = 0.85, *P* = 1.1 × 10^−11^), *ENST00000527943* (log_2_FC = 1.48, *P* = 8.1 × 10^−7^) and *ENST00000533795* (log_2_FC = 0.73, *P* = 0.009) demonstrated increased usage in high-osteoarthritis grade cartilage, while *ENST00000483129* (log_2_FC = −0.95, *P* = 2.2 × 10^−11^) usage was decreased (Fig. 5B). *ENST00000471714* is the longest transcript of *ABI3BP* and encodes the longest protein isoform (1786 aa), which contains two fibronectin III domains, important for structural and functional properties including integrin interactions, cell adhesion and the extracellular matrix (38). *ENST00000483129*, *ENST00000527943* and *ENST00000533795* encode shorter proteins (187, 151, 187 aa) that lack both 5′ and 3′ untranslated regions (UTRs) and the fibronectin III domain. The upregulated isoforms in high-osteoarthritis grade cartilage contain disordered regions spanning the first 105aa for *ENST00000527943*, all 187aa for *ENST00000533795* and amino acids 315–1529 for *ENST00000471714* (23)*.* We also detected differential usage for two transcripts of the *AKT1S1* gene. Transcript *ENST00000391834* (log_2_FC = 0.42, *P* = 0.008) demonstrated increased usage in high-osteoarthritis grade cartilage, while *ENST00000344175* (log_2_FC = −0.52, *P* = 3.3 × 10^−4^) usage was decreased (Fig. 5C). Although both transcripts code for the same protein, *ENST00000391834* has a longer 5′ UTR, which may affect its conformation and subsequently its translational regulation (39). Closer examination of the 5′ UTR of both *ATKT1S1* transcripts through functional diversity analysis (40) revealed that *ENST00000344175* contains an upstream open reading frame spanning positions 4–93, which is absent in *ENST00000391834.* Upstream open reading frame (uORFs) have been extensively linked to translation disruption in disease (41) and therefore their presence in *AKT1S1*, which is a repressor of the mTOR pathway, can have significant implications in mTOR subtle regulation. Another notable example was the isoform switching detected for *TRPM4* gene between its protein coding *ENST00000599628* transcript (log_2_FC = −0.83, *P* = 0.03) and its *ENST00000594568* (log_2_FC = 0.79, *P* = 0.03) transcript including a retained intron and not coding for any protein (Fig. 5D). Functional diversity analysis for these transcripts revealed that *ENST00000599628* contains multiple Alu elements along its coding region (at positions 933–1237, 1238–1375, 1388–1690) as well as a particular type of conserved interspersed repeat region called Mammalian-wide interspersed repeat (position: 592–765). These regions have been proposed to be associated with enhancer function and tissue-specific expression (42). *TRPM4* codes for a transient receptor potential ion channel activated by calcium. These type of channels have been reported to be involved in mechanotransduction (43) and have been proposed as a drug target for osteoarthritis (44).

Of the 82 genes showing differential transcript usage, none was found to be DE highlighting the additional level of information gained by transcript-level analysis. The genes showing differential transcript usage (isoform-only genes) were enriched for Gene Ontology (GO) terms including cadherin binding and cell adhesion at 5% FDR (Supplementary Material, Table S7).

### Differential splicing in osteoarthritis cartilage

Variety at the protein-transcript level is accomplished by a combination of events that include alternative transcription initiation and termination sites, splicing and polyadenylation (22). We investigated differential splicing phenomena and detected 230 differentially spliced junctions between low- and high-osteoarthritis grade cartilage in 209 genes (at 5% FDR). Of the 230 significant events, 157 included one or more novel exon junctions (Supplementary Material, Tables S8–S11). The leading signals based on the significance (FDR < 0.05) and their effect size (difference in the percentage of spliced in intron cassettes-|ΔPSI|) included *ABI3BP*, *COL11A1* and *S100A4* for which we identified four, two and one significantly differentially spliced junctions, respectively (Supplementary Material, Figs S2 and S4). For *ABI3BP* we detected increased inclusion of the exon cassette spanning the region (chr3:100808235–100846372) in high-osteoarthritis grade cartilage. This region is annotated to code the disordered parts of the *ABI3BP* protein and could potentially affect its structural conformation or binding state (45). For *COL11A1*, complex splicing phenomena, including decreased skipping of its eighth exon (Fig. 6), affect its N-terminal variable region (exons 6–9, chr1:103021769–103025521). As this region is present in all five *COL11A1* transcripts, it could have a regulatory role in the shape and size of the collagen fiber. Alternative splicing was also detected for other collagen coding genes, including *COL1A1*, *COL1A2* and *COL2A1*. For *COL1A1*, *COL1A2* and *COL2A1*, complex splicing phenomena, which include skipping of multiple exons, affect the coding region for the alpha1 chain of triple-helix collagen (Supplementary Material, Fig. S3). This domain is an important structural feature of collagen and a binding site for receptors, proteases and extracellular matrix proteins (46). With regard to *S100A4*, we identified increased skipping of the region chr1:153547846–153548345 spanning the first intron of *S100A4* and the second intron of *S100A3* (Supplementary Material, Fig. S4). This region includes a promoter-enhancer-like signature (EH38E1385470/GH01J153539) sequence which targets *S100A4*–*S100A5*. For the same gene, we also detected decreased skipping of the region (chr1:153544809–153545753) which also overlaps with another promoter region (EH38E1385466/GH01J153539). Differential splicing was also found for genes coding for regulatory components including *MIR22HG*, a lncRNA promoting osteogenic differentiation of mesenchymal cells through the PTEN/AKT pathway (47). We detected increased skipping of the third exon in all *MIR22HG* isoforms in high-osteoarthritis grade cartilage. We identified seven genes (*ABI3BP*, *PRDX1*, *SQSTM1*, *GADD45A*, *HNRNPM*, *PTPRE* and *CALR*) that showed evidence of both differential transcript usage and differential splicing between low- and high-osteoarthritis grade cartilage, suggesting that local splicing phenomena could underpin the observed different transcript usage for these genes. Differentially spliced genes were enriched for terms related to the extracellular matrix including proteoglycan and glycosaminoglycan binding, and cell surface interactions at 5% FDR (Supplementary Material, Table S11).

### Replication

We replicated our findings at gene level using an independent RNA-seq dataset from the RAAK study, which contained matched low- and high-osteoarthritis grade cartilage tissue from 17 knees of osteoarthritis patients (12) (Supplementary Material, Table S1). Comparing the effect sizes (log-fold-changes) of discovery and replication datasets, we found a high correlation between the effect sizes of DE genes (Pearson’s correlation *r* = 0.92, *P* < 10^−15^). This correlation was lower when considering all expressed genes (*r* = 0.36), indicating a higher level of noise for measurements of small and null effect sizes. In total, we detected 175 genes that were nominally significant in the discovery dataset and were replicated in the validation dataset (Supplementary Material, Table S12) and 38 genes being DE in both datasets (with |log_2_FC| > 1 and *P* < 0.05) (Fig. 7). Further examination of these genes indicated that they were enriched for known pathways related to osteoarthritis progression including serine/threonine protein kinase signaling (48), BMP signalling (49) and interleukin-6 signalling (50). All of these pathways have been proposed as drug targets for osteoarthritis (51–53) (Supplementary Material, Fig. S5B). Additional comparison of the 38 genes with the 16 studies reporting osteoarthritis-specific changes (4–19) in cartilage showed that each of the genes was found to be DE in at least another two studies. Hierarchical clustering analysis on the expression of the 38 genes indicated clustering based on the cartilage tissue grade (Supplementary Material, Fig. S5A). This suggests that the 38 genes accurately reflect transcriptomics changes in osteoarthritis cartilage supported by multiple lines of evidence and can be potentially interesting therapeutic targets.

## Discussion

We comprehensively assessed transcriptome-wide changes in osteoarthritis patient knee cartilage tissue in the largest patient cohort to date, and identified novel molecular markers of disease that robustly replicate in an independent dataset. Our key novel findings were the identification of differences in transcript usage for a total of 82 genes, genome-wide significant splicing differences between low- and high-osteoarthritis grade cartilage for 209 genes and the implication of 25 lncRNA genes among the 54 genes associated with osteoarthritis in knee cartilage for the first time.

Consistent with previous studies, we identified altered gene expression in biological pathways reflecting extensive remodelling of the extracellular matrix in an inflammatory environment (5,6,54). Specifically, we identified increased expression of members of interleukin-1 and interleukin-6 among generally increased cytokine activity and integrin surface interactions. Cytokines have been previously implicated in osteoarthritis progression as a secretion product of synovial macrophages being activated by fragments of extracellular matrix components. Recent studies have also shown that activated macrophages are polarized into either M1 or M2 populations with the M1/M2 ratio being higher in osteoarthritis synovium compared to controls (55). Additionally, M1 macrophages produce large amounts of inflammatory cytokines including IL-1 and IL-6, altering the chondrocyte environment and inducing also secretion of interleukins from chondrocytes (56). The increased expression of the M1 macrophage markers in high-osteoarthritis grade cartilage along with the significant upregulation of pro-inflammatory cytokines (*IL36B*, *IL11B*, *IL1B*) and lower *IL10* expression is in agreement with this model, indicating macrophage and chondrocyte interaction during osteoarthritis development.

With regard to extracellular matrix remodelling, we identified increased integrin interactions in high-osteoarthritis grade cartilage with osteopontin (*SPP1*) being the most upregulated integrin. Osteopontin is a multifunctional matricellular phosphoprotein with cytokine chemoattractant activities that mediates biomineralization, has been shown *in vitro* to promote the formation of calcium crystals in articular cartilage, and is associated with osteoarthritis severity (57,58). The significant upregulation of osteopontin in our study can therefore be indicative of increased calcification of high-osteoarthritis grade cartilage. Integrins are important connection molecules between the extracellular matrix components and their upregulation in our study can be potentially explained by the extensive reorganization of these components (mainly collagen) during osteoarthtis progression.

We identified downregulation for terms relevant to the translation machinery and ribosome biogenesis. More detailed view of these terms implicated the suppression of genes codding for the ribosomal small and large subunits as well as rRNA processing. This finding is in agreement with a recent model proposing that osteoarthritis can be also viewed as acquired ribosomopathy (59).

Our work extends our current understanding of the molecular events associated with osteoarthritis progression in that the depth of sequencing allowed detailed examination of changes beyond differential gene expression to include isoform-switching and splicing events. The lack of evidence for differential expression for the genes demonstrating differential transcript usage indicates additional signatures independent of gene expression. We report isoform-switches in genes relevant to osteoarthritis pathophysiology. These include *ABI3BP*, coding for a constituent protein of the extracellular matrix (38), which undergoes extensive remodeling in osteoarthritis (60), *AKT1S1*, coding for a repressor of mTOR, which is a proposed drug target for osteoarthritis (52), and *TPRM4* coding for a transient receptor ion channel activated by intracellular calcium levels (61), which are proposed to be altered in osteoarthritis chondrocytes as a response to mechanical stimuli (44).

We identified enrichment for cadherin binding among genes demonstrating isoform-switching. Cadherins are proteins that mediate cell–cell interactions, cell condensation and signaling in cartilage tissue (N and E-Cadherin) (62). Cadherin-11, in particular, has been previously associated with increased migration and invasive capacity of fibroblast-like synoviocytes, a cell population involved in osteoarthritis cartilage degradation (62). The above examples indicate the complementary nature of the transcript-level analysis and its relevance to osteoarthritis molecular mechanisms.

We also identified 209 genes with evidence of differential splicing between low- and high-osteoarthritis grade cartilage. Seven of these genes (*ABI3BP*, *CALR*, *PRDX1*, *SQSTM1*, *PTPRE*, *GADD45A* and *HNRNPM*) also showed differential transcript usage. The lack of a larger overlap can be attributed to the fact that transcript-level quantifications need to be imputed from short-read RNA-seq data using existing genomic annotations in contrast to local splicing. Therefore, these estimates are affected by limitations of short-read sequencing (63) as well as by the fact that transcriptomic diversity is not fully represented in the reference transcriptome. Additionally, our analysis did not include detection of phenomena of intron retention, which was the case for 10% of the cases of differentially used transcripts between low- and high-osteoarthritis grade cartilage. A potential biological explanation can also be that other mechanisms, such as the use of alternative transcription start sites, alternative termination sites and polyadenylation may explain better the differences between low- and high-osteoarthritis grade cartilage compared to splicing (22). Further, splicing can simultaneously affect multiple transcripts of the same gene without changing their expression. For example, we found decreased skipping of the eighth exon for *COL11A1* (among the complex splicing events affecting the N-terminal protein region) affecting all of its transcripts in high-grade cartilage. As the N-terminal contains regulatory sequences including heparan sulfate proteoglycan binding motifs, alternative splicing could affect the interaction with extracellular matrix constituents, including glycoproteins and collagen type II (64). In addition to that, we identified differential inclusion of promoter sequences for *S100A4. S100A4* has been found to be upregulated in osteoarthritis chondrocytes (also in our study log_2_FC = 0.8, *P* = 1.9 × 10^−15^) and has been proposed to be a transcriptional regulator of *MMP13*, which is a key metalloprotease of extracellular matrix degradation (65)*.*

Cohort size coupled to sequencing depth also allowed the identification of further DE genes including the less abundant lncRNAs. To our knowledge, we are the first to report differences in lncRNA expression in knee osteoarthritis cartilage at this depth using RNA-sequencing, utilizing >100 million reads per sample, which is five times the depth of all RNA-seq studies published to date exploring lncRNAs in knee osteoarthritis cartilage (9,11,18,19). *MYOSLID*, which demonstrated the largest increase in high-osteoarthritis grade cartilage, is in a positive feedback loop with the transforming growth factor (TGF)-β/SMAD, a pathway involved in osteoarthritis development through regulation of articular chondrocyte hypertrophy and maturation (31,66). Furthermore, it has been shown that antisense lnRNAs can function in *cis* on their overlapping protein-coding genes affecting their expression. It has also been demonstrated that lncRNAs can have enhancer-like functions leading to increased expression of their protein coding target genes (67). This may be the case for *TENM3-AS1*, one of the leading upregulated lncRNAs in high-osteoarthritis grade cartilage, due to the presence of enhancer regulatory elements along its sequence (23) and the consistent upregulation of its sense *TENM3* gene. Another potential explanation can also be that *TENM3* and *TENM3-AS1* are co-expressed due to their proximity and the lack of autonomy observed between neighboring genes in gene expression (68). *TENM3* encodes for Teneurin 3, a transmembrane protein, a mutation which has been associated with hip dysplasia (69). *TENM3* has previously been found to be upregulated in high-grade cartilage (4–6,8). To understand *TENM3-AS1* mechanism of action and its potential clinical relevance in the modulation of *TENM3* levels, further functional studies are warranted.

In order to gain a better understanding of the potential function of lncRNA, we calculated their enrichment among lncRNA annotation datasets and identified enrichment among 166 RBP. To identify the potential interactions in osteoarthritis cartilage we focused on the RNA-biding proteins that were also DE in our dataset and calculated their correlation of expression with the DE lncRNAs. The highest positive correlations were found for the pairs *PPARG*-*TENM3-AS1* and *PPARG-LINC01411. PPARG,* which was found significantly upregulated in our study consistent with previous RNA-seq studies comparing low- and high-osteoarthritis grade cartilage (4–6,8), encodes for a ligand-activated transcription factor with a chondroprotective role and has been proposed as a therapeutic target for osteoarthritis through activation of mTOR/autophagy pathway (37). *LINC01411* has been reported to be the most upregulated lncRNA in the study of lncRNA in osteoarthritis from Hoolwerf *et al*. (11), as well as in our study. *TENM3-AS1* is a lncRNA identified first time in our study. Since both *LINC01411* and *TENM3-AS1* are located in different chromosomes than *PPARG,* it is likely that they regulate *PPARG* in *trans*.

Our study has limitations. We compared low- and high-osteoarthritis grade cartilage in patients with end-stage disease. Therefore, the observed transcriptional differences may differ from molecular changes involved in early stage disease. Additionally, a common mechanism of action of lncRNAs includes ‘sponging’ of microRNAs and reducing their availability to target mRNAs (33). To this end obtaining microRNA expression data using small RNA-seq would increase the functional interpretation of the associated lncRNAs. Quantifications on the transcript level were based on short-read RNA-seq data and, therefore, factors that include incomplete annotation, coverage and GC content could affect quantification estimate accuracy. To this end, long-read sequencing would achieve more accurate transcript estimates. Going forward, the study of protein-level data will extend the biological relevance of the findings to give a direct quantitative measure of the different protein isoforms on cartilage tissue.

In conclusion, we used deep sequencing in the largest knee osteoarthritis population to progress our understanding of the genomics of the disease. In addition to the discovery of differential gene expression signatures associated with known and new biological pathways in osteoarthritis, our analyses revealed qualitative and quantitative differences in transcript usage and splicing across multiple genes that are also associated with disease severity independent of differential gene expression. Our analysis also revealed potential interactions between RBPs and lncRNA including *TENM3-AS1* and *LINC01411,* which can be potential preclinical targets by modulating *PPARG* expression levels. These findings increase our understanding of the biological complexity that hallmarks the disease. Further in-depth examination of these transcriptional variations through perturbation experiments will help unravel their role in the causal pathways of osteoarthritis and the tissue specificity of the observed variants.

## Materials and Methods

### Study samples

Patients undergoing knee replacement for osteoarthritis with no history of significant knee surgery (apart from meniscectomy), knee infection, or fracture and no malignancy within the previous 5 years were recruited. We further confirmed that no patient had been treated with corticosteroids (systemic or intra-articular) within the previous 6 months, or any other drug associated with immune modulation. Both within-patient, matched cartilage samples were taken from the weight-bearing parts of the joint to ensure biomechanical loading did not influence within-pair differences in gene expression and were scored macroscopically using the International Cartilage Repair Society (ICRS) scoring system (70). From each patient, we obtained one cartilage sample of ICRS grade 0 or 1 signifying low-osteoarthritis grade degeneration (‘low-grade sample’) and one sample of ICRS grade 3 or 4 signifying high-osteoarthritis grade degeneration (‘high-grade sample’). All study participants provided informed consent and samples were collected under Human Tissue Authority license 12182 and National Research Ethics Service approval 15/SC/0132, South Yorkshire and North Derbyshire Musculoskeletal Biobank, University of Sheffield, UK. Cohorts’ ethical approval for the RAAK study collection was obtained from the medical ethics committee of the LUMC under protocol numbers P08.239 and P19.013.

This project was conducted under a National Research Ethics Service approved biobank that is overseen by a steering committee, which includes two lay members. The lay members reviewed this project proposal prior to its initiation, and had the opportunity to comment upon and make edits to the study design, as did the Sheffield Lay Advisory Panel for Bone Research. The conduct of the biobank and its outputs are also reviewed by the biobank lay committee members.

### RNA sequencing and preprocessing

RNA was extracted using Qiagen AllPrep RNA Mini Kit, as per manufacturer’s instructions and previously described in Steinberg *et al*. (4). Poly-A tailed RNA (mRNA) was isolated from total RNA using Illumina’s TruSeq RNA Sample Prep v2 kits. RNA was fragmented and libraries were prepared and multiplexed according to standard Illumina protocols. The libraries were sequenced on the Illumina HiSeq 2000 and HiSeq 4000 (75 bp paired-ends), yielding a median of 115 million reads per sample (IQR: 104.4–132.7). We used Samtools v1.10.2 (71) and biobambam v2.0.148 (72) to convert files from the compressed cram format to the fastq format. We performed quasi-mapping of the reads on the reference transcriptome Ensembl GRCh38 release 97 (cDNA and non-coding) (23) using Salmon v.14 (73), yielding a median of 89.5% (IQR: 87.2–91.2) of reads mapped and 74.8% (IQR: 71.5–77.8) of de-duplicated reads. All the analyses after generation of the count matrix were performed using R v.3.6.1 (74) and Bioconductor v.3.10 (75).

Gene abundances were quantified using tximport using the same reference (76). The transcript-level expression estimates were summarized to gene-level length scaled transcripts per million (TPM) estimates by specifying countsFromAbundance = lengthScaledTPM option. For the differential transcript usage analysis, the transcript-level estimates were scaled to TPM estimates by specifying txOUT = TRUE and countsFromAbundance = dtuScaledTPM options in tximport. We excluded genes and transcripts with low expression levels (<1 count per million in >50 samples for genes and in >90 samples for transcripts). These thresholds were defined based on the voom (77) plot exploring the mean–variance trend for different expression thresholds. The optimal threshold was selected visually as the one of removing genes with low expression without a drop in the variance.

Samples that had a mapping percentage <75% (12 samples), had two or more FastQC (78) fails (six samples) and poor RNA quality measured (RNA Integrity Number <5; 22 samples) were excluded from the analysis. We also excluded samples based on non-European ancestry of individuals (six samples) and abnormal expression density plots (seven samples). For individuals with bilateral knee replacement, we excluded one pair of matched samples each (eight samples), keeping only the sample pair with the best quality. Finally, the analysis was restricted to paired low-and high-osteoarthritis grade cartilage samples. The final dataset included 15.872 genes and 35.140 transcripts for 248 paired low- and high-osteoarthritis grade cartilage samples from 124 osteoarthritis patients.

For the differential splicing analysis, we aligned reads to the reference genome GRCh38 release 97 (23) using the STAR v2.7.6a (79) two-pass mode and including the XS strand tags to all canonically spliced alignments based on their intron motifs (parameters: alignSJoverhangMin = 8, outSAMstrandField = intronMotif). The resulting Binary Alignment Map (BAM) files were converted to juncfiles using Regtools v0.5.2.6a (80) (regtools junctions extract command) specifying an 8 nt anchor length and a 50 and 50.000 nt minimum and maximum intron length, respectively.

### Identification of surrogate variables representing hidden confounders

We used surrogate variable analysis to enable adjustment for hidden confounders and unwanted technical variation. SVAseq (81) yielded 14 surrogate variables separately for gene-level and transcript-level summarized matrices. These surrogate variables were included as covariates when testing gene-level expression differences and differential transcript usage between low- and high-osteoarthritis grade cartilage.

### Differential gene expression

We tested differential gene expression between low- and high-osteoarthritis grade cartilage using limma (82) using filtered counts which were also normalized for observational-level weights after mean variance estimation (voom transformation) (77) to remove heteroscedasticity. The final model accounted for the paired study design and for the 14 surrogate variables representing technical variation. We used the Benjamini–Hochberg False Discovery Rate (FDR) to correct for multiple testing. We defined DE as the genes that had FDR < 5% and a fold change more than 2 in either direction (|log_2_FC| > 1) (83). The biotypes of the DE genes were extracted using the Bioconductor package AnnotationHub (84). Comparison of the results of differential expression with another 16 RNA-sequencing and microarray studies containing more than five patients were performed after summarizing their respective results to the Ensembl gene stable IDs (4–19).

### Differential transcript usage

For each gene, we tested for differences in transcript usage using median normalized transcript abundances. Differences in transcript usage were computed using (82) diffSplice function using voom (77) transformed counts. Expression of each transcript was compared to the average expression of all other transcripts of the same gene in a series of *t*-tests. Raw *P*-values were aggregated to the gene level using the function perGeneQValue from the DEXSeq package (85). The stage-wise method implemented in the stageR (86) package was applied to the raw *P*-values to control the gene-level false discovery rate. Transcripts with stage-wise adjusted (OFDR) *P*-values ≤0.05 were considered significant. The relative transcript usage for each transcript was calculated by diving the batch corrected counts of the transcript of interest by the total counts of the all the transcripts belonging to the same gene customizing the function plotDTU from satuRn package (87). Plots were created using ggplot2 (88). Individual transcript features (Number of exons, presence of 3/5′ UTR regions) were extracted from the Ensembl version 97 (23) .gtf file (Homo_sapiens.GRCh38.97.gtf) using functions from GenomicFeatures package (89). To evaluate the regulatory potential and functional consequences of the differentially used transcripts we performed functional diversity analysis implemented in tappAS software (version 1.0.7) (40).

### Differential local splicing analysis

Local splicing changes were assessed using Leafcutter v.0.2.9 (90), which quantifies differential intron usage across samples. Initially, intron clustering was performed using Binary Alignment Map (BAM) files output of alignment using STAR v2.7.6a (79). Variably spliced introns were called using all samples (total of 248 paired low- and high-osteoarthritis grade cartilage samples—same as for all the other analyses). Differential splicing was assessed using a Dirichlet-multinomial generalized linear model. The final model included the condition of interest (low- versus high-osteoarthritis grade cartilage), the paired sample status and the estimated unknown technical variation in the form of 14 surrogate variables (same covariates as used for differential transcript usage). The overlapping introns (spliced reads) were clustered using the default parameters. We specified 50 split reads supporting each cluster and allowed introns of up to 500 kb. This resulted in 44 589 successfully called intron clusters belonging to 13350 genes. Intron clusters with FDR < 5% were considered significant. Significant intron clusters were visualized using the Leafviz shiny app. The individual differential splicing events were further manually inspected for exon skipping, alternative exon usage, alternative 5′ or 3′ site usage and complex splicing. The differentially spliced clusters were mapped to transcripts using the GViz package (91). The function proteinToGenome from ensembldb package (92) was used to map protein domains on the transcripts (93). The different splicing events were manually inspected. The splicing events discussed in the text were the ones that combined the highest statistical significance and effect size (measured in percent spliced in for each intron cluster—ΔPSI) of at least 4%.

### Functional enrichment

Gene set enrichment analysis and overrepresentation analyses were performed using the Molecular Signatures Database (MSigDB v7.0) gene collections including GO (C5—Biological Process-BP and Molecular Function-MF), Kyoto Encyclopaedia of Gene and Genome (KEGG) (C2), REACTOME (C2) and transcription binding motifs (C3) annotations (94,95). For characterization of DE genes, we used GSEA ranking genes by their effect size (log-fold change) and enrichr and GSEA functions from clusterProfiler (96). In order to get a better understanding of the enriched terms, we calculated the gene semantic similarity between the enriched terms using the Jaccard coefficient (96) and performed hierarchical clustering of the terms using the ward.D2 clustering algorithm. Additionally, we performed GSEA using the curated markers of human cell types. The CellMarker data were downloaded from http://bio-bigdata.hrbmu.edu.cn/CellMarker/ (97).

Due to the lack of representation of lncRNA genes in GO and KEGG, functional enrichments for lncRNA genes were tested separately using the annotation datasets and overrepresentation analysis provided by the platform LncSEA, which contains information for downstream targets of lncRNAs (34). Specifically, DE lncRNAs between low- and high-osteoarthritis grade cartilage were tested for overrepresentation among the RBP sets from the LncSEA database (34).

For genes showing evidence of differential splicing or differential transcript usage, we applied overrepresentation analysis. In case a gene had more than one significant intron clusters, we considered the differential splicing result of the most significant cluster and the background list included all genes with identified intron clusters. For GO, KEGG and REACTOME analyses, we only considered terms annotated to >10 and <250 genes in order to avoid inaccuracies in the calculation of normalized enrichment scores as proposed by GSEA authors (95). Significance for the enriched pathways was defined at 5% FDR.

### LncRNA–RNA-binding protein potential interactions

As lncRNA expression is highly tissue-specific (98) we have built upon the proposed targets from overrepresentation analyses and identified the ones that are DE in our dataset and significantly co-expressed with DE lncRNAs in osteoarthritis cartilage. Specifically, in order to detect potential regulation of lncRNAs by the RBP coding genes, we calculated the Spearman correlation between the identified DE lncRNAs and DE RBP genes in our dataset. LncRNA expression data was normalized using log(CPM), and the batch effect in the form of surrogate variables was removed using the limma removeBatchEffect function (82). Spearman’s correlations were calculated using the Hmisc R package (version 4.2.0). Correlations with *P*-values less than 0.05 were considered significant. Network visualization was performed using Cytoscape v.3.8.2 (99).

### Replication

The differential expression analysis results were validated in an independent dataset containing paired low- and high-osteoarthritis grade cartilage tissue from participants of the RAAK study (*n* = 17 patients after QC) (12) (Supplementary Material, Table S1). The replication dataset was analyzed using the same approach and software as the discovery dataset, with small adaptations to reflect the smaller number as samples as follows: reads that had a mapping percentage <50%, had >2 FastQC (78) fails or had abnormal gene expression density plots as described for the discovery dataset were excluded from the analysis. Genes with low expression levels in the replication dataset (<1 CPM for >10 samples) were excluded. We defined DE genes in the replication dataset as those that had a larger than 2-fold (|log_2_FC| > 1) change in high-osteoarthritis grade cartilage at 5% FDR. The comparison of replication results to the discovery results was done using the transcriptome-wide FDR adjusted *P*-values for both replication and discovery datasets. Hierarchical clustering was performed using robustly replicated genes (|log_2_FC| > 1 and FDR < 0.05 in both datasets). For clustering samples, covariate-adjusted logCPM values were used with ward.D2 hierarchical clustering algorithm and Spearman clustering distance.

## Supplementary Material

Supplemetary_figure_1_ddac017

Supplemetary_figure_2_ddac017

Supplementary_figure_3_ddac017

Supplementary_figure_4_ddac017

Supplementary_figure_5_ddac017

Supplementary_table_1_ddac017

Supplementary_table_2_ddac017

Supplementary_table_3_ddac017

Supplementary_table_4_ddac017

Supplementary_table_5_ddac017

Supplementary_table_6_ddac017

Supplementary_table_7_ddac017

Supplementary_table_8_ddac017

Supplementary_table_9_ddac017

Supplementary_table_10_ddac017

Supplementary_table_11_ddac017

Supplementary_table_12_ddac017

## Data Availability

Summary statistics of all analyses will be shared through the Musculoskeletal Knowledge Portal (mskkp.org) upon publication. Raw data have been deposited to the European Genome/Phenome Archive under the following accession numbers: EGAD00001005215, EGAD00001003355, EGAD00001003354, EGAD00001001331.
