# Supplementary figures and images for: A molecular map of long non-coding RNA expression, isoform switching and alternative splicing in osteoarthritis
Source: Hum Mol Genet. 2022 Jan 28;31(12):2090–105. doi: 10.1093/hmg/ddac017 (PMC9239745; doi:10.1093/hmg/ddac017)

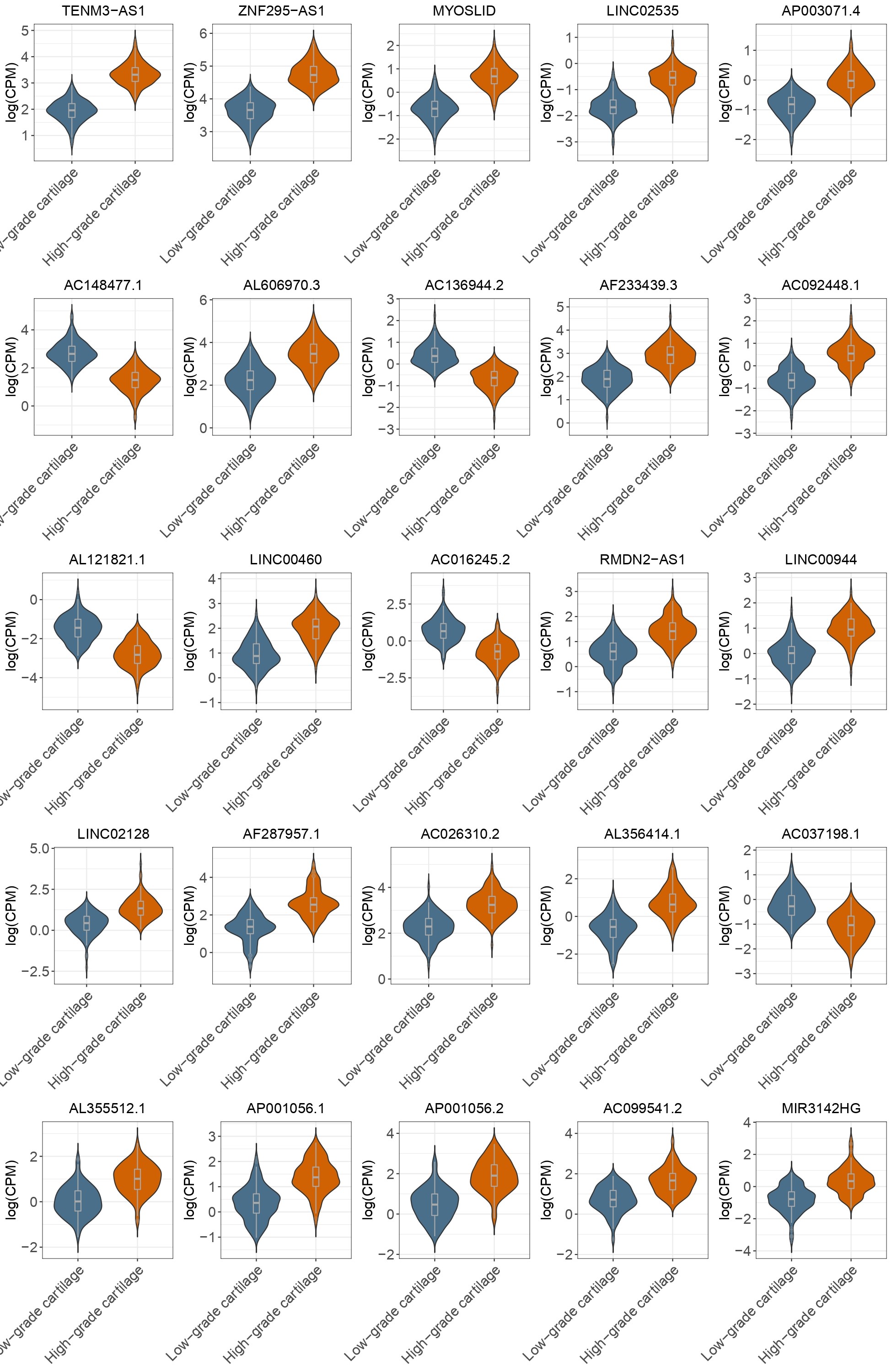

Supplement: Supplemetary_figure_1_ddac017 [file supplemetary_figure_1_ddac017.jpeg]

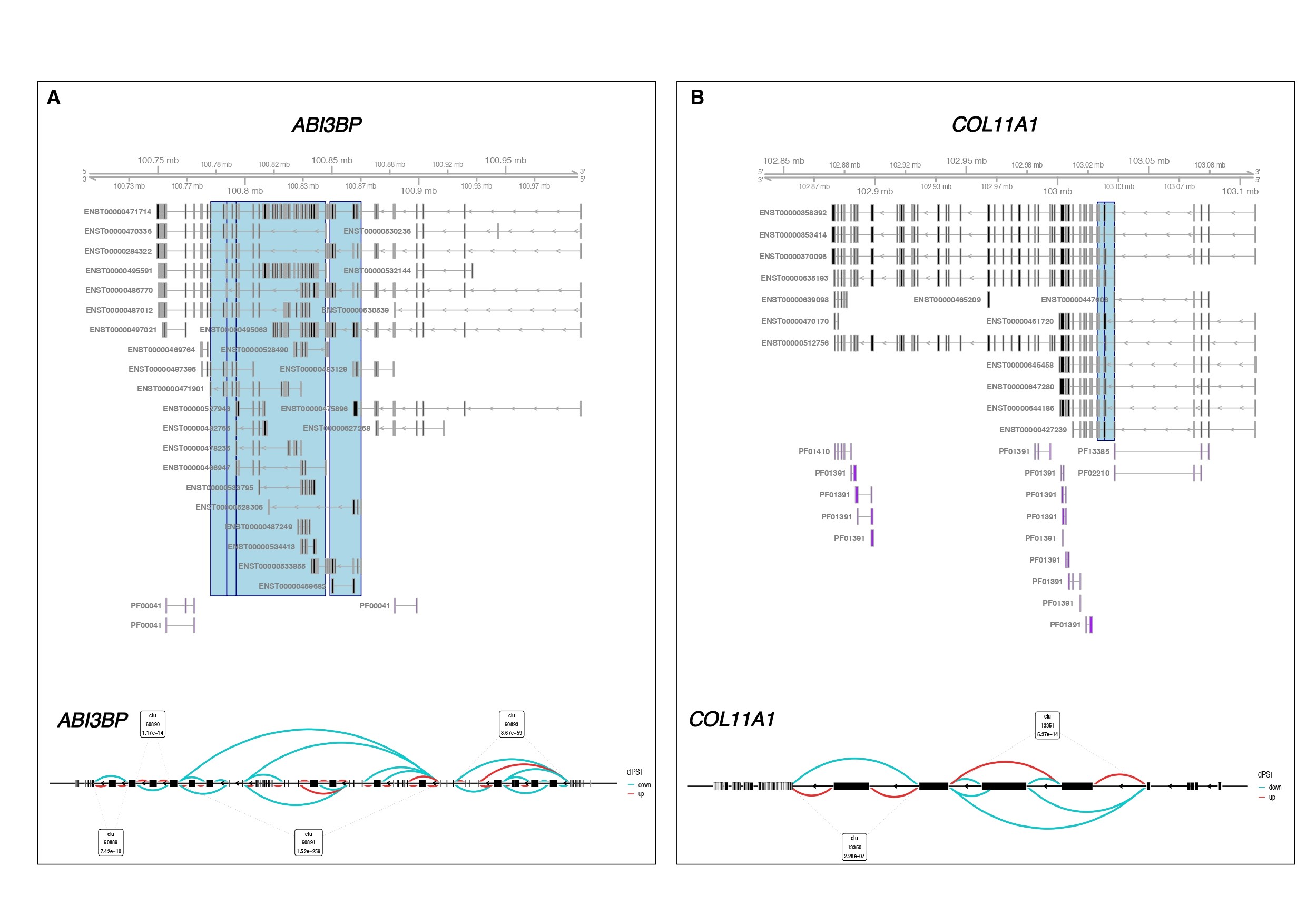

Supplement: Supplemetary_figure_2_ddac017 [file supplemetary_figure_2_ddac017.jpeg]

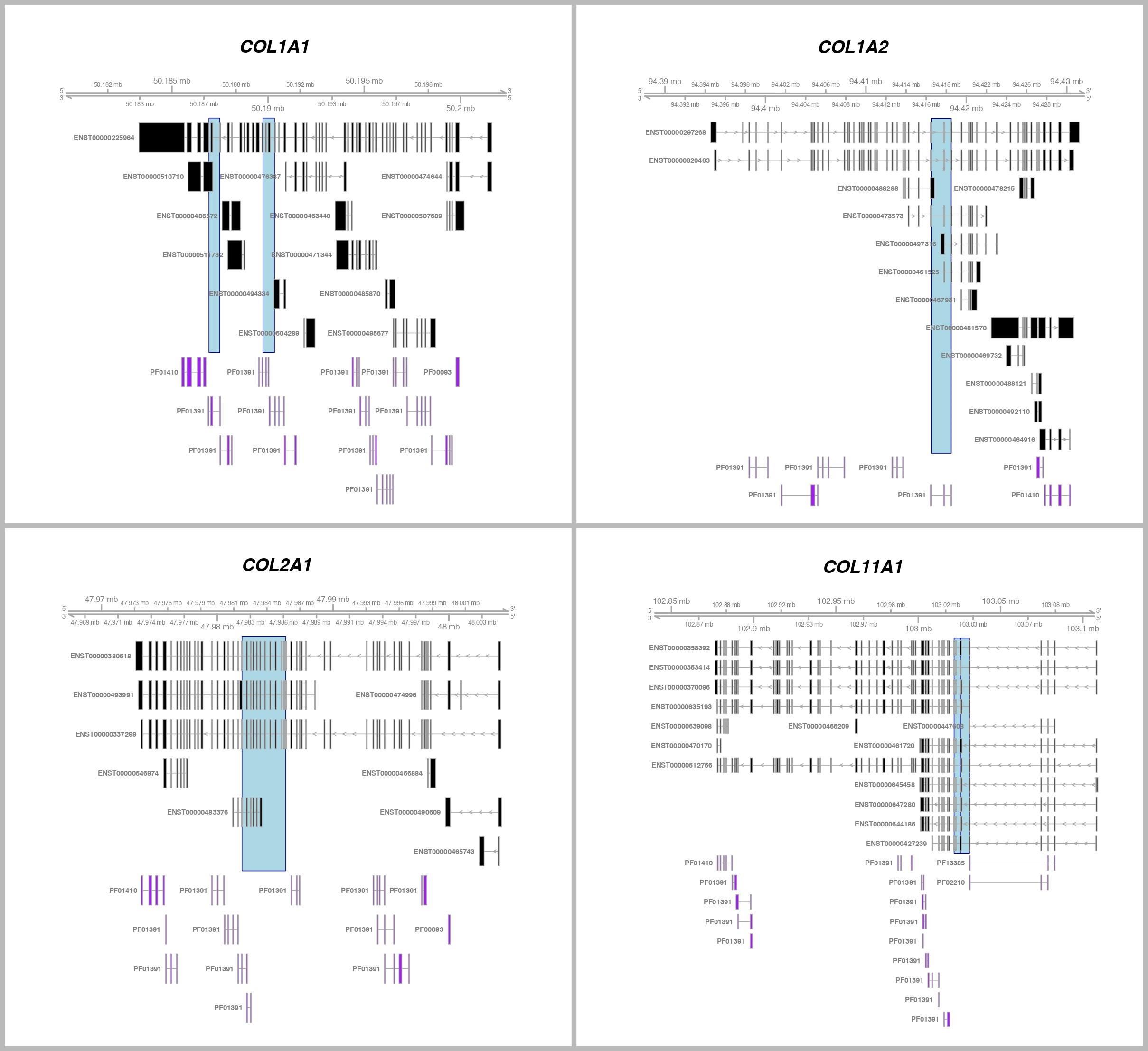

Supplement: Supplementary_figure_3_ddac017 [file supplementary_figure_3_ddac017.jpeg]

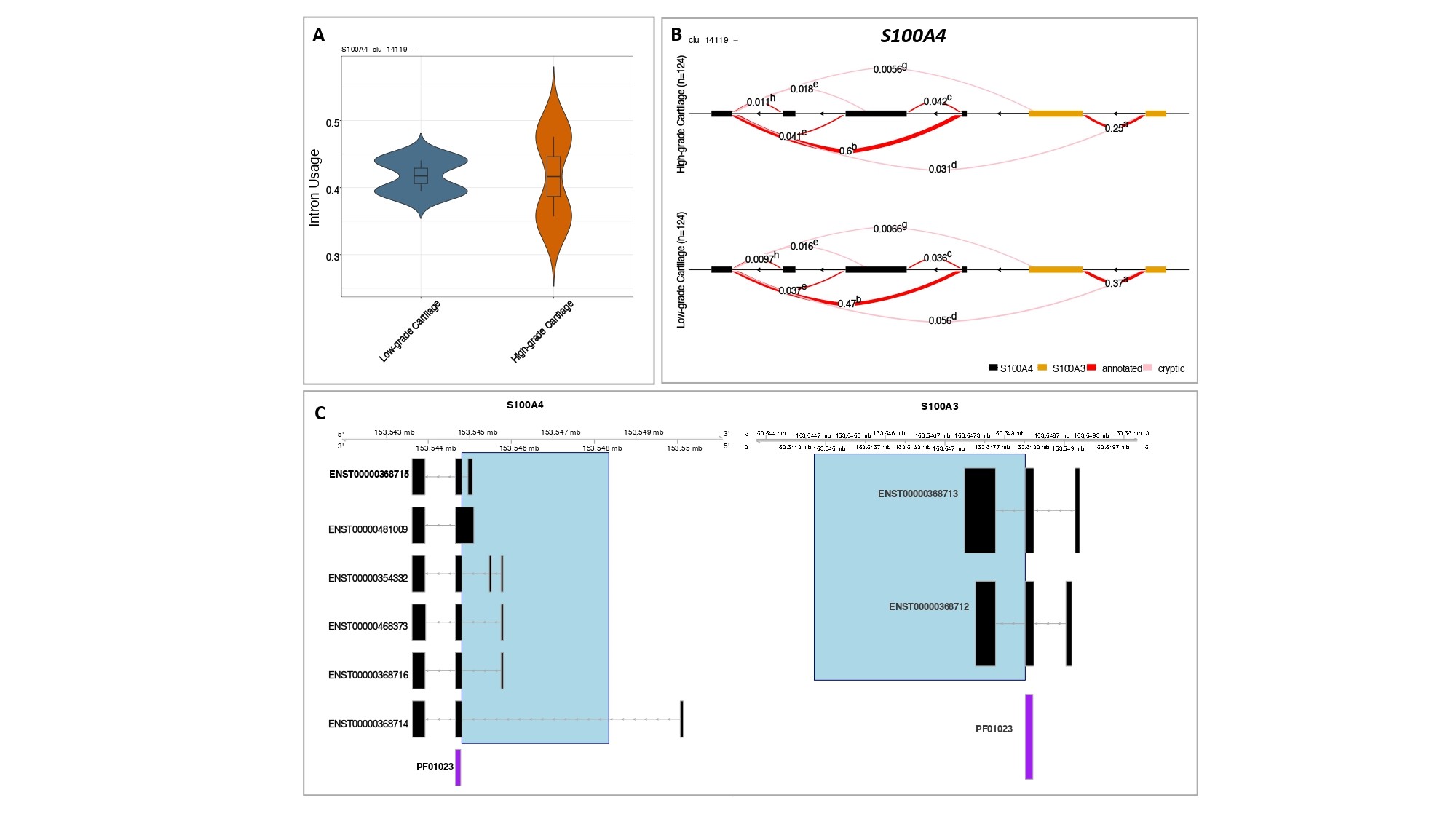

Supplement: Supplementary_figure_4_ddac017 [file supplementary_figure_4_ddac017.jpeg]

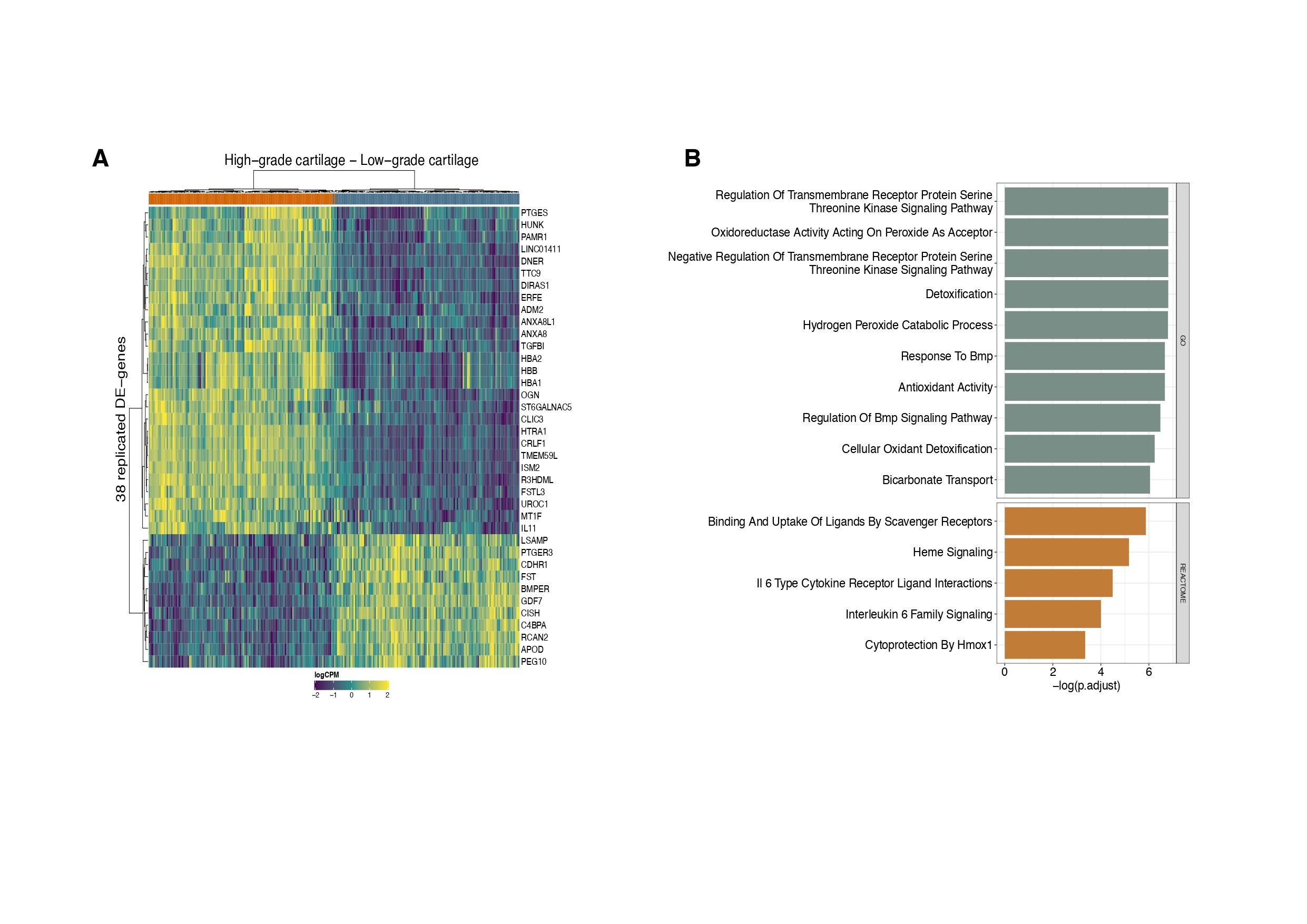

Supplement: Supplementary_figure_5_ddac017 [file supplementary_figure_5_ddac017.jpeg]
